# American trypanosomiasis, or Chagas disease, in Panama: a chronological synopsis of ecological and epidemiological research

**DOI:** 10.1186/s13071-017-2380-5

**Published:** 2017-10-10

**Authors:** Indra G. Rodriguez, Jose R. Loaiza

**Affiliations:** 1 Instituto de Investigaciones Científicas & Servicios de Alta Tecnología, Ciudad del Saber, República de Panamá; 2 Smithsonian Tropical Research Institute, Balboa Ancón, Republic of Panama; 3 Programa Centroamericano de Maestría en Entomología, Universidad de Panamá, Panamá, República de Panamá

**Keywords:** *Trypanosoma cruzi*, Triatominae vectors, Animal reservoirs, Transmission cycles, Community ecology, Control

## Abstract

American trypanosomiasis, or Chagas disease, is a growing public health problem in Panama, and further forest degradation due to human population growth is expected to worsen the situation. Most people infected with the parasite *Trypanosoma cruzi* are silently ill, and their life expectancy is severely compromised, which contributes to further deterioration of living conditions in endemic regions. Here, we review the outcomes of nearly 100 years of ecological and epidemiological investigation about Chagas disease in Panama, in an attempt to highlight progress, identify needs, and re-orient future efforts. *Rhodnius pallescens* and *Triatoma dimidiata* are both primary vectors of *T. cruzi* in Panama, but *R. pallescens* seems more efficient in human-altered forest ecosystems due to a greater degree of association with *Attalea butyracea*. In contrast, *T. dimidiata* transmits *T. cruzi* efficiently under more sylvatic conditions (e.g. settlements inside old-growth or secondary forest patches), where its populations reach considerable numbers irrespective of the absence of *A. butyracea*. A trend of increasing forest degradation, suburbanization, and development of tourism in Panama favoring the establishment of *A. butyracea* and other palm tree species (*Acrocomia* sp.) suggests that a colonist species like *R. pallescens* will continue to play a more prominent role in the transmission of *T. cruzi* than a forest specialist like *T. dimidiata*. However, studies about the taxonomic status and ecology of these vectors are still needed in Panama to address their transmission potential fully. The implementation of an active surveillance system and education programs could greatly minimize the risk of Chagas disease transmission in Panama, preventing fatal infections in children from endemic areas.

**Electronic supplementary material:**

The online version of this article (10.1186/s13071-017-2380-5) contains supplementary material, which is available to authorized users.

## Background

American trypanosomiasis is a human parasitic infection caused by the protozoan *Trypanosoma cruzi* (Eucarya, Kinetoplastea, Trypanosomatidae). The infection, known as Chagas disease in honor of its discoverer, Carlos R. J. Chagas [1], is a zoonosis affecting a wide range of wildlife vertebrates, which spread to humans primarily by kissing-bug invertebrates [2–5]. In the Americas, 21 countries are considered endemic, including Panama, and between six and 12 million people are infected, mostly in Latin America [6–8]. In Panama, *Trypanosoma rangeli* is also known to infect humans, but its public health significance is negligible compared to that of *T. cruzi*. Other species of trypanosomes existing in the country that do not infect humans are *Trypanosoma forattini*, *Trypanosoma hippicum*, *Trypanosoma vivax*, *Trypanosoma theileri* and *T. cruzi cruzi* (*s*.*l*.) (Table 1) [9–15].

**Table 1:** List of vertebrate hosts and reservoirs for trypanosome species in Panama

Host order	Host family	Host species	Common names (English/Spanish)	Trypanosome species	Reference	Geographical origin	Diagnostic test
Chiroptera	Phyllostomidae	*Desmodus rotundus* (Geoffroy, 1810)	vampire bat/vampiro común	*T. hippicum*	[9]	Former Panama Canal Zone (Panama)	UND
Artiodactyla	Bovidae	*Bos taurus* Linnaeus, 1758	cattle/ganado vacuno	*T. theileri*	[10]	UND	UND
Carnivora	Canidae	*Canis lupus familiaris* Linnaeus, 1758	dog/perro doméstico	*T. cruzi*	[17]	Former Panama Canal Zone (Panama)	Examination of blood by the thick film method; inoculation of guinea pigs, mice and rats with blood taken from infected dogs
Cingulata	Dasypodidae	*Dasypus novemcinctus* Peters, 1864	nine-banded armadillo/armadillo	*T. cruzi*	[17]	Arraiján (Panama), Patuga and Parita (Herrera)	Examination of blood by the thick film method
Didelphimorfia	Didelphidae	*Didelphis marsupialis* Linnaeus, 1758	opossum or black-eared opossum/zarigüeya común	*T. cruzi*, *T. rangeli*	[17, 24]	Chilibrillo Caves (Panama), Alhajuela (Panama), Parita and Patuga (Herrera)	Examination of blood by the thick film method
Rodentia	Sciuridae	*Sciurus gerardi morulus* Bangs, 1898	squirrel/ardilla	*T. cruzi*	[17]	Former Panama Canal Zone (Panama)	Examination of blood by the thick film method
Chiroptera	Phyllostomidae	*Hemiderma perspicillatum aztecum* (Saussure, 1869)	short tail bat/murciélago de cola corta	*T. cruzi*	[17]	Chilibrillo Caves and Bella Vista (Panama)	Examination of blood by the thick film method; inoculation of laboratory animals
Chiroptera	Phyllostomidae	*Phyllostomus hastatus panamensis* Allen, 1904	spear-nosed bat/murciélago de nariz lanceolada	*T. cruzi*	[17]	Chilibrillo Caves (Panama)	
Chiroptera	Phyllostomidae	*Uroderma bilobatum* Peters, 1866	tent-making bat/murciélago de orejas	*T. cruzi*	[17]	Summit (Panama)	
Chiroptera	Phyllostomidae	*Glossophaga soricina leachi* Gray, 1844	pallas’s long-tongued bat/murciélago siricotero	*T. cruzi*	[17]	Bella Vista near Miraflores Locks (Panama)	
Chiroptera	Phyllostomidae	*Atribeus jamaicensis jamaicensis* Leach, 1821	fruit bat/murciélago zapotero	*T. cruzi*	[17]	Summit Zoo (Panama)	
Artiodactyla	Bovidae	*Bos taurus* Linnaeus, 1758	cattle/ganado vacuno	*T. vivax*	[11]	Aguadulce (Coclé)	Examination of thick blood smears
Didelphimorfia	Didelphidae	*Caluromys derbianus* (Waterhouse, 1841)	derby’s woolly opossum/comadreja	*T. cruzi*	[24]	UND	Examination of thick blood smears
Didelphimorfia	Didelphidae	*Philander opossum* (Linnaeus, 1758)	gray four-eyed opossum/zorra cuatro ojos	*T. cruzi*	[24]	UND	
Chiroptera	Phyllostomidae	*Desmodus rotundus* (Geoffroy, 1810)	vampire bat/vampiro común	*T. cruzi*	[24]	UND	
Chiroptera	Phyllostomidae	*Carollia perspicillata* (Linnaeus, 1758)	seba’s short-tailed bat/murciélago carolia	*T. cruzi*	[24]	UND	
Primates	Cebidae	*Cebus capucinus* (Linnaeus, 1758)	white-fronted capuchin/mono cariblanco	*T. cruzi, T. rangeli, T. minasense*	[24] ^b^	Alanje and Barú (Chiriquí), Darién and Panama	Examination of thick and thin blood smears stained with Giemsa; inoculation into hemoculture tubes; direct microscopical examination
Primates	Atelidae	*Ateles fusciceps* Gray, 1866	black-headed spider monkey/yerre	*T. cruzi*	[24] ^b^	Chepo, Panama and Darién	
Primates	Callitrichidae	*Saguinus geoffroyi* (Pucheran, 1845)	the Panamanian, red-crested or rufous-naped tamarin/mono tití	*T. cruzi, T. rangeli, T. minasense*	[24, 90] ^b^	Panama and Colón	
Primates	Cebidae	*Saimiri sciureus* (Reinhardt, 1872)	common squirrel monkey/mono ardilla	*T. cruzi*	[24] ^b^	Chiriquí	
Primates	Cebidae	*Alouatta villosa* (Gray, 1845)	red howler monkey/mono aullador	*T. mycetae*	b	La Chorrera, Chepo, Panama, Darien and Los Santos	
Primates	Aotidae	*Aotus trivirgatus* (Humboldt, 1812)	three-striped night monkey/mono nocturno	*T. sp.*	b	La Chorrera, Capira, Arraiján, Panama, Colón and Darién	
Pilosa	Myrmecophagidae	*Tamandua tetradactyla* (Linnaeus, 1758)	collared anteater or lesser anteater/oso hormiguero	*T. cruzi*, *T. rangeli, T. legeri*	[24, 84]	Panama	Examination of fresh blood films; inoculation of culture media
Pilosa	Megalonychidae	*Choleopus hoffmanni* Peters, 1858	two-toed sloth/perezoso de dos dedos	*T. cruzi*	[24]	UND	Examination of thick blood smears
Pilosa	Bradypodidae	*Bradypus variegatus infuscatus* Wagler, 1831	three-toed sloth/perezoso de tres dedos	*T. cruzi*	[24]	UND	
Rodentia	Sciuridae	*Sciurus granatensis* Humboldt, 1811	red-tailed squirrel/ardilla colorada	*T. cruzi*	[24]	UND	
Rodentia	Echimyidae	*Proechimys semispinosus* Tomes, 1860	tome’s spiny rat/mocangué	*T. cruzi*	[24]	UND	
Rodentia	Dasyproctidae	*Dasyprocta punctata* Gray, 1842	agouti or common agouti/neque	*T. cruzi*	[24]	UND	
Rodentia	Muridae	*Rattus rattus* (Linnaeus, 1758)	black rat/rata negra de los tejados	*T. cruzi*	[24] ^b^	UND	
Rodentia	Cricetidae	*Tylomys panamesis* (Gray, 1873)	panamanian climbing rat/rata trepadora	*T. cruzi*	[24]	UND	
Rodentia	Echimyidae	*Diplomys labilis* (Bangs, 1901)	rufous tree rat/rata espinosa	*T. cruzi*	[24]	UND	
Carnivora	Procyonidae	*Nasua narica* (Linnaeus, 1766)	white-nosed coati/gato solo	*T. cruzi*	[24]	UND	
Carnivora	Procyonidae	*Potos flavus* (Schreber, 1774)	kinkajou/cusumbi o mico de noche	*T. cruzi*	[24]	UND	
Carnivora	Procyonidae	*Bassaricyon gabbii* Allen, 1876	bushy-tailed olingo/olingo	*T. cruzi*	[24]	UND	
Rodentia	Muridae	*Mus musculus* Linnaeus, 1758^a^	mouse/ratón	*T. rangeli*	[24]	UND	
Rodentia	Muridae	*Ratus norvegicus* (Berkenhout, 1769)^a^	rat/rata	*T. rangeli*	[24]	UND	
Rodentia	Cricetidae	*Oryzomys capito* (Olfers, 1818)	large-headed rice rat/rata arrocera	*T. forattinii*	[12]	Trinidad forest (Panama)	Examination of heart blood smears
Squamata	Phyllodactylidae	*Tetradactylus rapicauda* (Houttuyn, 1782)	turnip-tailed gecko/gecko	*T. thecadactyli*	b	UND	Examination of toe blood smears or brachial artery stained using Giemsa technique

^a^Experimentally infected with *T. cruzi*

^b^Additional file 1: Table S1

Undetermined: UND

**Fig. 1:**
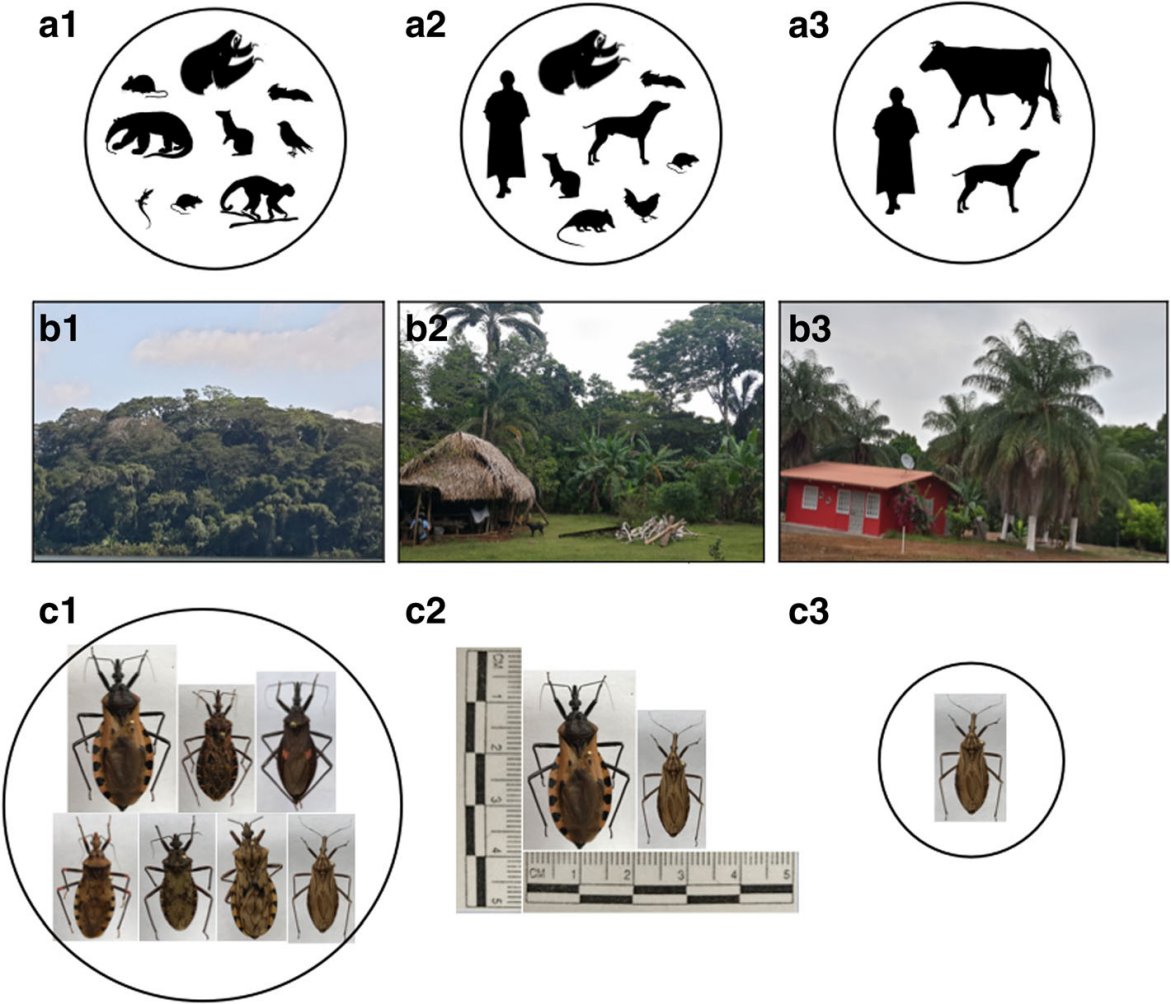
Eco-epidemiological transmission patterns of *Trypanosoma cruzi* in Panama: **a1-a3** Animal hosts and/or reservoirs of *T. cruzi* across a gradient of forest habitat degradation. Main reservoirs *Didelphis marsupialis* (opossum), *Choloepus hoffmanni* (two-toed sloth), and *Bradypus infuscatus* (three-toed sloth); secondary reservoirs *Proechymis semispinosus* (prickly rat), *Dasypus novemcinctus* (armadillo), *Tamandua tetradactyla* (Anteater), *Artibeus jamaicensis*, *Rattus rattus* (common rat), *Dasyprocta punctata* (agouti) and *Canis familiaris* (dog). **b1** Endemic transmission of *T. cruzi* at sylvatic enzootic foci (accidental transmission in humans). **b2** Epidemic transmission in forest-altered habitats (indigenous communities). **b3** Sporadic transmission in highly altered habitats (farmers “Campesinos”). **c1**-**c3** Triatomine bugs vectors of *T. cruzi* across a gradient of natural forest habitat degradation. **c1** top row, left to right, *Triatoma dimidiata*, *Triatoma dispar*, *Eratyrus cuspidatus*: bottom row *Pastrongylus geniculatus*, *Pastrongylus rufotuberculatus*, *Pastrongylus humeralis*, *Rhodnius pallescens*. **c2** *Triatoma dimidiata*, and *Rhodnius pallescens*. **c3** *Rhodnius pallescens*

Research into epidemiological aspects of Chagas disease (herein CHD), including ecological factors, began in 1930 with the first case study published by Miller [16]. Initially, researchers did not have accurate diagnostic tools to identify the trypanosome species responsible for causing infection in humans. Therefore, scientific articles at the time referred to a parasite similar in morphology to *T. cruzi* [17–20]. In 1937, Johnson & Kelser [21] published the first epidemiological study about CHD in Panama; this effort looked at the incidence of human trypanosomes in endemic regions using an immunological test. However, during the following years, there were no related publications. Scientific efforts on CHD increased in Panama toward the end of the 1950s, with the seminal work of Dr. Octavio Sousa on the biology and ecology of triatomine bugs. Dr. Sousa reported three species of triatomines naturally infected with *T. cruzi*, worked on the development of diagnostic methods for *T. cruzi*, and investigated the distribution of *T. cruzi* and *T. rangeli* in endemic areas of Panama [22, 23]. In addition, Sousa identified a preliminary list of vertebrate hosts and reservoirs for these two trypanosome species (Table 1, Fig. 1) [13, 24–27]. As a result, efforts by Dr. Sousa contributed to a better understanding of CHD in southern Central America. Other researchers added important contributions between 1970 and 1990, which were largely about parasite biology, biochemistry, pathogenesis and treatment of CHD itself [28–34]. The focus of these investigations was on the transmission cycle, the taxonomy of insect vectors, and the identification of animal reservoirs of *T. cruzi* [35–40]. More recently, important scientific advances were made in the detection and identification of *T. cruzi* and *T. rangeli* using serological and molecular techniques [41–43], plus additional CHD foci were discovered in rural areas of Panama [44, 45].

The scope of eco-epidemiologic research about CHD in Panama has evolved through time. For most of the twentieth-century, scientific efforts adopted a pattern of discovery and data reporting type of research, which greatly helped to unravel the natural history of this complex zoonotic disease. At present, however, the focus is centered on studies trying to understand the community ecology of CHD [46–48]. Chagas disease, as with many other multilayered zoonotic diseases, requires a community-scale approach to complement traditional epidemiological approaches to untangle disease transmission. The proposed synthesis of “disease community ecology” offers a theoretical framework and the analytical tools to move beyond clinical outcomes of disease in humans, and considers the full suite of species that influence infection dynamics [48]. Moreover, in this more holistic conceptual framework, not only is the classic epidemiological triad considered (i.e. parasite-vector-host), which was widely studied between 1960 and 1990, but also the influence of habitat alteration on disease prevalence [2, 46–48]. Recent efforts in Panama investigated the impact of anthropogenic habitat alterations on the community structure of hosts and vectors and assessed mechanisms by which these changes may increase transmission risk. Gottdenker and colleagues [49, 50] applied community ecology as the framework, and hypothesis testing as the method, to understand how habitat fragmentation affects the interactions between parasites, vectors, and reservoirs comprising the enzootic cycle of CHD.

Lately, there has been a growing regional interest to review information about vector-borne infectious diseases affecting humans. These efforts are key to effectively manage neglected tropical zoonotic infections such as *Plasmodium vivax* malaria, leishmaniasis, and CHD, among others [3–5, 7, 51]. The information conveyed in these documents could be used to design efficacious prevention and mitigation strategies targeting the pathogens that cause these infections. The information can also help to appreciate the array of studies completed locally about these zoonoses, thus helping to avoid redundant research pursuits and to steer the scientific agenda further. The rationale for this review article is to describe the historical evolvement of scientific research about the ecology and epidemiology of CHD in Panama. In so doing, we aim at highlighting the work of prominent researchers and their key findings in studies conducted since 1930 (Additional file 1: Table S1). We put special emphasis on the transmission dynamics of *T. cruzi*, the bionomic of some species of Triatominae vectors, and the role of habitat degradation into transmission risk. Our considerations may potentially help to identify research needs and to re-orient future efforts about what is currently considered to be a growing public health concern in Panama.

### Symptomatology, diagnosis and treatment

CHD is a two-phase clinical infection; the acute phase progresses in individuals of all ages, but children are typically most affected [24, 33, 34]. During the acute phase, a unilateral palpebral edema and conjunctivitis with ipsilateral regional lymphadenopathy, known as Romaña sign, develop around the eyebolt. However, many Panamanian patients do not have this symptom [24, 52]. Fatal cases during the acute phase of CHD in Panama are characterized by severe dyspnea and progressive myocarditis with arrhythmia, cardiomegaly, vomiting, and anuria. After the acute phase, infected people enter an indeterminate phase without symptomatology (i.e. chronic phase), but are still considered ill with positive serology [24, 50, 53, 54]. The most common clinical manifestations in confirmed cases of CHD in Panama are cardiac arrhythmia, cardiomegaly, dysfunction of nerve conduction, fever, and cervical and submaxillary adenitis. However, patients in both phases may have no apparent clinical symptoms [16, 19, 34].

In the Southern Cone region (southern South America), CHD is frequently associated with megaviscera (i.e. megaesophagus and megacolon), in Panama, several studies of esophageal transit failed to detect these manifestations in local patients. Parasitemia is short, and the chronic phase is benign in Panama compared to the one documented in South America [24, 28, 55, 56]. Some studies suggest that different clinical manifestations between Panama and South American countries could be the result of genetic divergence among discrete geographic strains of *T. cruzi*, or due to differences in human immune responses. Furthermore, similar clinical manifestations to those found in Panamanian patients were also detected in *Rattus rattus* (common rat) and *Canis familiaris* (domestic dog), which were either infected with *T. cruzi* naturally or experimentally [20, 30, 57]. These findings may indicate the existence of a distinct strain of *T. cruzi* in Panama, which may harbor specific phenotypic features regarding pathogenicity and virulence. This theory would explain the distinct clinical profile found locally. Scientific studies in Panama have confirmed the existence of a discrete strain of *T. cruzi*, which is thought to have co-evolved intimately with *Didelphis marsupialis* (black-eared or common opossum) and *Rhodnius pallescens* [58–61]. This strain is known as *T. cruzi* I, and it is genetically different from South American strains [58, 59, 62]. Currently, *T. cruzi* is subdivided into six discrete typing units (DTU) (i.e. TcI, TcII, TcIII, TcIV, TcV and TcVI), of which TcI is the most widely distributed in the Americas. Furthermore, there is empirical evidence suggesting that TcI transits between sylvatic and domestic cycles and that it is associated with cardiac complications in humans [63–65].

Very few CHD cases were diagnosed in Panama at the time of its discovery [16, 19]. In fact, detection of *T. cruzi* is rare in the country thus far, even with better diagnostic methods, and people often visit hospitals for other reasons without knowing they are infected. Johnson & Kelser [21] attributed this difficulty to the irregular occurrence of parasites in peripheral blood and to the lack of accuracy in microscopic examination intended to identify *T. cruzi*. Also, inoculation and culture of *T. cruzi* in susceptible animals or xenodiagnostic techniques using insect vectors are limited approaches to detect low volumes of parasites in the blood. Johnson & Kelser [21, 66] demonstrated the presence of trypanosomes throughout most of Panama. These authors inspected 10,570 human samples for the presence of trypanosomatids using the fixation complement test and estimated an overall infection rate of 1.86%. However, most positive samples came from the former Panama Canal Zone. The fixation complement test, based on cultures of *T. cruzi*, was considered an adequate diagnostic tool at the time because it did not display cross-reaction among different species of trypanosomes. Later on, other studies provided serological and biochemical markers for the detection of *T. cruzi*, and these have been widely used since the 1970s [22, 24, 67]. More recently, significant advances were made in parasite diagnosis and identification through molecular techniques such isoenzyme genotyping, multiplex PCR, and automated Sanger DNA sequencing for nuclear loci [43, 60, 68]. Because of reagent- and equipment-related costs, these modern molecular techniques are expensive, whereas rapid serological tests are cost-effective and useful tools to diagnose *T. cruzi* infections in humans. However, there is still a concern about false-positive results due to cross-reactivity with *T. rangeli* [2].

There are very few reports about treating or curing CHD in Panama [54]. The first clinical cases reported in the country were managed without medication [16]. In 1976, Blandón et al. [33] administered doses of metronidazole, primaquine, levofuraltadone, and isopentaquina to 47 patients, including 44 in the acute phase. Metronidazole was the most effective drug because it successfully cleared the infection without patient intolerance or death. Currently, the most used drugs to treat *T. cruzi* infections in Panama are nifurtimox [5-nitrofuran (3-methyl-4-(5′-nitrofurfurylideneamine)tetrahydro-4H-1,4-tiazine-1,1-dioxide] and benznidazole [2-nitroimidazole (N-benzyl-2-nitroimidazole acetamide]. These drugs were developed four decades ago, have limited efficacy in patients in the chronic phase, and can produce harmful side effects [2, 54, 69–72]. Recent advances in drug discovery in Panama, specifically bioactive components extracted from the bacterium *Bacillus pumilus*, which was isolated from the black coral *Antipathes* sp., have shown the ability to constrain the growth of *T. cruzi* [73]. Other compounds derived from Panamanian isolates of the mangrove *Pelliciera rhizophorae* have also demonstrated selective anti-parasitic activity against *T. cruzi* [74]. It is worth mentioning that drug trials in Panama using bioactive components extracted from bacteria, corals, and other trypanostatics have all been in vitro. Thus, there are still many steps before they can be considered in human trials.

### Eco-epidemiology and transmission

Conventionally, *T. cruzi* infection in Panama has been typified as a forest zoonosis, with humans being casually infected when entering the enzootic cycle, which is disseminated to a great variety of animal vertebrates, by six species of blood-sucking triatomine bugs (Fig. 1). Notwithstanding, transmission can proceed along a gradient of forest degradation, not in a spillover fashion like in the case of arthropod-borne viral pathogens (i.e. arboviruses) and mosquitoes, but rather as a well-adapted system in which deforestation fosters biodiversity losses while boosting the ecologic links between primary triatomine vectors and major animal reservoirs of *T. cruzi* [49, 50]. Moreover, deforestation, urbanization, and other human activities can bring people into closer contact with triatomine disease-carrying vectors, thus creating opportunities for the colonization and establishment of these insects in human settlements [4]. However, not every kind of landscape change seems to increase CHD epidemiologic risk in Panama. Rather, transmission of *T. cruzi* in human-altered settings appears to be worsened by artisanal practices that use certain palm trees and their foliage and fruits for food, to build thatch roofs of houses or to make fermented wine-like beverages [75, 76]. Because of this type of exploitation, these palms proliferate abundantly throughout rural topographies of the country, and serve as a disease epicenter, favoring the aggregation of key vectors and reservoirs of *T. cruzi* near human habitations (Fig. 1). Research in Brazil suggested that the reproductive biology of some species of *Attalea* benefit the most from environmental changes such as deforestation and soil degradation [77].

*Trypanosoma cruzi* and *T. rangeli* are often found co-infecting animals or humans; both species are endemic to the neotropics and co-occur within Panama [22, 24]. Transmission of *T. cruzi* occurs during blood consumption when triatomine bugs deposit trypomastigote forms along with their feces near the bite site. Later, affected individuals scratch this area, dragging parasites into the wound or eyes, thus facilitating the invasion of internal tissues [2]. *Trypanosoma rangeli*, on the contrary, is transmitted via saliva when bugs are ingesting blood [78, 79]. The infection caused by *T. rangeli*, reported for the first time in Panama in 1957, is symptomless due to low pathogenicity compared to the infection with *T. cruzi* [22]. *Trypanosoma rangeli* is usually found infecting the digestive tract, hemolymph, and salivary glands of *R. pallescens*, but in Panama, it does not seem to be capable of infecting other bugs, including *Triatoma dimidiata* [24]. This implies that a high degree of specificity exists between the Panamanian strain of *T. rangeli* and *R. pallescens* [78, 79]. Other ways of transmission of *T. cruzi* and *T. rangeli* to humans, such as organ transplants or blood transfusion from infected donors, although possible, have not been reported in Panama.

CHD, mainly detected in the provinces of Panama, Coclé and Colon, seems largely localized to central Panama, where human infection rate normally ranges from 0.5 to 8.8% [22]. However, since adequate ecologic conditions for the transmission of *T. cruzi* have been reported from the entire country, this is likely due to the concentration of studies in this area, plus considerable case is underreporting nationwide [22, 24, 49, 80]. Sousa & Johnson [22] reported *T. rangeli* to be six times more prevalent in rural Provinces of Panama, Coclé and Colón than *T. cruzi*, based on microscopic examination. Sousa [24] speculated that this was due to a steady contact between people and *R. pallescens* since this vector is the only one capable of transmitting *T. rangeli* in Panama. However, *R. pallescens* is found infected with *T. cruzi* more frequently than with *T. rangeli*, which suggests differences in the vectorial competence of *R. pallescens* for these two parasite species [22, 42, 81]. The greater prevalence of *T. rangeli* in humans could be due to a more efficient way of transmission via saliva as compared to that of *T. cruzi* via contaminated feces [3]. Some studies also suggest that this outcome is likely due to the fact that an initial exposure to *T. rangeli* might confer immune protection against a subsequent infection with *T. cruzi* [22, 42, 81, 82]. Recently, new CHD foci were detected in the district of Santa Fe, located north of Veraguas’s Province, and also in Chepo and Chiman, in eastern Panama. Authors in these studies reported equivalent infection rates of *T. cruzi* and *T. rangeli* in humans as well as in *R. pallescens* [44, 45].

### Animal reservoirs

CHD is in principle a neotropical zoonosis that involves a large variety of vertebrate and invertebrate species as hosts, reservoirs, and vectors. However, due to increased international travel in recent decades, this infection has greatly expanded from its original geographical range [2–4]. In 1972, Octavio Sousa [24] published a list of 26 species of mammals in Panama that were found infected with *T. cruzi* based on microscopy. This record included six species of rodents, five bats, four primates, three marsupials, edentates, and carnivores, in that order (Table 1; Fig. 1). Among the rodents and marsupials, *R. rattus* and *D. marsupialis* are considered major reservoirs of *T. cruzi* in peridomestic settings, whereas *Proechimys semispinosus* (prickly rat) is an important reservoir under sylvatic conditions [30, 83]. Other species of wild animals that serve as a reservoir of *T. cruzi* in Panama are *Dasypus novemcinctus* (armadillo), *Tamandua tetradactyla* (anteater), *Bradypus infuscatus* (three-toed sloth), and the bat *Artibeus jamaicensis* [17, 84, 85].

Birds in general, including chickens, are considered refractory to infection with *T. cruzi*, which might suggest that they could be good candidates for zoo-prophylactic control strategies [86]. Besides, chickens frequently eat triatomines in peridomiciliary and domiciliary areas and could diminish their populations to some degree. For example, Cecere et al. [87] proposed that the exclusion of chickens from peridomiciliary areas could increase *T. cruzi* infection rates in humans. Moreover, recent studies conducted in rural localities of central Panama reported dogs commonly infected with *T. cruzi*, reaching prevalence rates of up to 11.1%, which could also suggest a role as a domestic reservoir [88, 89].

*Trypanosoma rangeli* has been identified from 15 species of wild mammals including *D. marsupialis*, which is frequently found co-infected with *T. cruzi* (Table 1, Fig. 1) [17]. Sousa & Dawson [90] proposed *Saguinus geoffroyi* (titi monkey) as another natural reservoir of *T. cruzi* and *T. rangeli* in Panama and anticipated a high risk of infection to people adopting these animals as pets. These monkeys can migrate from nearby forested areas into houses, possibly searching for food. A high prevalence of *T. rangeli* in *S. geoffroyi* implies a close relationship with *R. pallescens*, but this could also be due to horizontal transmission during the rainy season when these monkeys feed massively on triatomine bugs [13]. Because of the pleomorphic nature of epimastigotes of *T. cruzi*, surveillance studies based on microscopy are insufficient to describe pathogen-host relationships. Follow-up studies, based on molecular approaches to identification (i.e. DNA barcoding), are needed to confirm the specificity of pathogen-host species associations.

### Vectors of *T. cruzi* and *T. rangeli*

Triatominae bugs vectoring *T. cruzi* in Panama were identified during the 1930s; these insects belong to various genera within the subfamily Triatominae (order Hemiptera), and are commonly known in Panama as “chinches mamones” or “chinches de monte” [35, 38, 83, 91]. Triatominae species found naturally infected and capable of transmitting *T. cruzi* in Panama are *Triatoma geniculata* (named as *Panstrongylus geniculatus* later on) [17, 18], *R. pallescens* [92], *Eratyrus cuspidatus* [93] and *T. dimidiata* [94] (Table 2). Clark & Dunn [17] incriminated *R. prolixus* as one of the main vectors of *T. cruzi* in Panama, but the occurrence of this species was never confirmed in the country [24, 95]. Furthermore, mistakenly identified as *Triatoma venosa* by Champion [96] and Usinger [97], *Triatoma dispar* is another potential vector of *T. cruzi* in Panama, and was found naturally infected in the forest canopy of eastern Panama [23]. In addition, *Panstrongylus humeralis* and *Panstrongylus rufotuberculatus* were incriminated as vectors of *T. cruzi* in Panama, but these are primarily sylvatic species associated with animal caves, burrow nests, and tree holes in pristine forest environments [25]. Therefore, they are not usually found near human settlements [24]. *Cavernicola pilosa*, a cave-dwelling species parasitizing bats, was also found infected with *T. cruzi* in Panama [24, 91].

**Table 2:** Triatominae species found naturally infected and capable of transmitting *T. cruzi* and *T. rangeli* in Panama

Triatominae taxa	Trypanosome species [ref]	Habitat	Host-feeding range [ref]	Geographical location [Province]	Sampling site	Ecotype	Methodology
*Panstrongylus geniculatus*	*T. cruzi* [17]	Sylvatic species specialized on subterranean host habitats: caves, nests and tree holes. It has been associated with the armadillo (*Dasypus novemcinctus*)	UND	Chilibrillo caves [Panama]	Bat cave	Sylvatic	Examination of bug feces; inoculation of guinea pigs with macerated bugs; feeding bugs on guinea pigs (xenodiagnostic technique)
*Rhodnius pallescens* s.l.	*T. cruzi* [92]; *T. rangeli* [79]	Anthropic colonist species, invades both indoor and outdoor environments depending on ecological, enviromental and soci-economic conditions. It is strongly associated with species of palm trees, e.g. *Attalea butyracea* ^a^ and also *Acrocomia* spp.	Bradypodidae, Cracidae, Didelphidae, Echimyidae, Myrmecophagidae, Sauria (Lizards), Sciuridae, [100]	Aguas Buenas [Panama]	Inside house	Domestic	Feeding bugs on guinea pigs (xenodiagnostic technique)
			Amphibia, Canidae, Cebidae, Columbidae, Cracidae, Cricetidae, Dasyproctidae, Didelphidae, Echimyidae, Felidae, Hominidae, Muridae, Leporidae, Phasianidae, Psittacidae, Reptilia, Rallidae, Suidae, Strigidae (Sauria), [37]				
			Birds, dogs, humans, opossum, rats [104]				
			Artiodactyla, Carnivora, Caudata, Chiroptera, Ciconiiformes, Falconiformes, Galliformes, Marsupialia, Passeriformes, Primata, Rodentia, Squamata, Xenarthra, [50]				
*Eratyrus cuspidatus*	*T. cruzi* [93]	Sylvatic species rarely found near human habitations in rural areas	UND	Retiro Matias Hernandez [Panama]	Inside building	Domestic	Feeding bugs on guinea pigs (xenodiagnostic technique)
*Triatoma dimidiata*	*T. cruzi* [94]	Sylvatic or semi-anthropic species found occasionally indoor, but mainly encountered around houses in domestic animal shelters	Accipitridae, Amphibia, Ardeidae, Bovidae, Canidae, Columbidae, Cricetidae, Felidae, Hominidae, Leporidae, Muridae, Passeriformes, Phasianidae, Psittacidae, [39]	Chorrera [Panama]	Inside house	Domestic	Feeding bugs on guinea pigs (xenodiagnostic technique)
*Cavernicola pilosa*	*T. cruz*i [91]	Sylvatic species associated with the caves inhabited by various species of bats	UND	Panama City [Panama]	Bat cave	Sylvatic	–
*Triatoma dispar*	*T. cruzi* [23]	Sylvatic species found in the canopy of mature old-growth type of forest	UND	Cerro Quia [Darien]	Forest canopy	Sylvatic	Examination of bug feces; inoculation of white mice with suspension of fecal material from bugs
*Panstrongylus rufotuberculatus*	*T. cruzi* [24]	Sylvatic species, specializes on subterranean host habitats: caves, nests and tree holes. It is often found in pristine seasonal tropical rainforest forest	UND	Cerro Quia [Darien]	Forest canopy	Sylvatic	Examination of bug feces; inoculation of white mice with suspension of fecal material from bugs
*Panstrongylus humeralis*	*T. cruzi* [25]	Sylvatic species that specializes on subterranean host habitats: caves, nests and tree holes. It has been found sporadically around houses in rural areas	UND	Bayano Lake [Panama]	Forest understory	Sylvatic	Inoculation of white mice with suspension of fecal material from bugs

^a^ Formerly known as *Scheela zonensis*

Reference: ref

Undetermined: UND

*Rhodnius pallescens* and *T. dimidiata* are considered to be the primary vectors of *T. cruzi* in Panama [22, 24, 35, 38, 76, 97]. The former appears to predominate in the central part of the country, whereas *T. dimidiata* is most commonly known from the western region [22, 24]. This apparent inter-species spatial segregation seems more associated with discrete environmental circumstances in these areas matching the ecologic requirements of each species. For instance, *R. pallescens* thrives in central Panama, where in the last 60 years human population growth has prompted the transformation of forest into land for agriculture and livestock production. These landscape changes favor the proliferation of certain palm tree species [76], which in turn, seem to facilitate the demographic expansion of *R. pallescens* [98]. In contrast, *T. dimidiata* appears to be more associated with less-altered forest habitats in western Panama, where it is able to maintain large and stable population sizes without palm trees [24, 99].

### Vector bionomics of *R. pallescens* and *T. dimidiata*

Pipkin [85] posited that ecological niche, the degree of domiciliation, host feeding behavior, and rate of infection with *T. cruzi* are the most important factors shaping the local transmission role of different species of triatomines. In Panama, *R. pallescens* is closely associated with *Attalea butyracea* (e.g. Royal, Wine, or Corozo palm), a species of palm tree formerly known as *Scheelea zonensis* [75, 99, 100]. *Attalea butyracea* is prevalent across the country, is found in both primary and secondary forest habitats, but most commonly in savanna ecosystems, prairies, and realms for agriculture and livestock development, often in close proximity to human habitations [75, 76, 100]. This palm offers proper conditions of humidity and temperature as well as food (i.e. *D. marsupialis*) for the development of *R. pallescens* [98, 100]. Moreover, recent studies suggested that *R. pallescens*, which under laboratory conditions can fly up to 5 km before tiring, could invade houses attracted by light from nearby palm trees [101, 102]. Therefore, the presence of *A. butyracea* near human communities is considered an important risk factor for CHD transmission.

Pipkin [85] deduced that the abundance of *R. pallescens* in houses of CHD endemic communities from central Panama exceeded that of other triatomines. He found nymphs and adults of *R. pallescens* inside houses, showing for the first time that this triatomine species could enter households and nourish on humans. However, in other studies, *R. pallescens* was collected most commonly outdoors than indoors [101, 103]. Differences in the degree of domiciliation of *R. pallescens* across Panama could be an artifact of ecological, demographic, and socioeconomic disparities among different study sites. For example, particular housing conditions are essential for the colonization and adaptation of *R. pallescens*; houses built with mud, clay, and palm leafs may be more vulnerable to invasion than those built with brick, cement, and metal roofs. Moreover, houses surrounded by palm trees (e.g. *Attalea* spp. and *Acrocomia* spp.) and animal shelters might promote a faster invasion of *R. pallescens* as opposed to others that lack these conditions [2, 3, 24, 100].

Initial studies about host-feeding ranges of Panamanian triatomines indicated that *R. pallescens* feeds mainly on *D. marsupialis* and humans, but it can also take blood from rodents, canines, felines, monkeys, reptiles, and wild**/**domestic birds (Table 2) [37, 100, 104]. Gottdenker et al. [49] proposed that host species spectra serving as food sources for *R. pallescens* vary as a function of habitat fragmentation, with *Choleopus hoffmanni* (i.e. two-toed sloth) being the primary host in areas of old-growth and secondary forests, and *D. marsupialis* being the primary host in forest-altered settings close to human settlements (Fig. 1). Some researchers consider *R. pallescens* to be a forest specialist in Panama, but its opportunistic feeding behavior and a remarkable capacity to invade and adapt to different environmental conditions (including human-related niches) [105] confer it an advantage over other triatomines. This capacity allows it to flourish in both sylvatic and peridomestic areas, where wildlife and humans are the main sources of blood, correspondingly [50]. Contrary to *R. pallescens*, *T. dimidiata* has not been commonly associated with palm trees, nor has it frequently been found indoors in Panama [45]. Christensen et al. [39] hypothesized that western populations of *T. dimidiata* feed mainly on humans, chickens, and dogs, but they do not seem to feed on *D. marsupialis*. However, some authors consider this finding to be erroneous and attribute it to low specificity by the precipitin test employed in previous studies. Although studies conducted in Panama provided a general view of the host-feeding ranges of *R. pallescens* and *T. dimidiata*, it is discernible that both species are catholic feeders that take blood from a large variety of vertebrates, probably depending on their availability and biomass, which in the peridomestic setting may be mostly rodents and humans [37, 104, 106, 107].

Sousa & Johnson [70] estimated *T. cruzi* infection rates of triatomines from central Panama to be between 3.1–21.5%. Vásquez et al. [103] used microscopy and reported 85.4 and 14.6% infection rates with *T. cruzi* and *T. rangeli* in *R. pallescens*, respectively. Calzada et al. [101] used molecular techniques and estimated the infection rates of *R. pallescens* with *T. cruzi* and *T. rangeli* at 72.7 and 40.0%, respectively. As expected, results obtained with molecular methods were superior to those obtained with microscopy, where only 27.3% of specimens tested positive for trypanosomes. In contrast, the infection rate of *T. dimidiata* with *T. cruzi* in Panama was significantly lower than that of *R. pallescens*, ranging between 13.5–17.7% [22, 45]. Gottdenker et al. [49, 50] suggested that the infection rate of *R. pallescens* with *T. cruzi* is influenced by the degree of habitat fragmentation, which in turn determines host species composition and availability. They reported a higher infection rate of *R. pallescens* with *T. cruzi* in deforested and fragmented forest sites compared with more contiguous and less altered forest habitats. Future studies aiming to investigate *T. cruzi* infection rates and host-feeding ranges in Panamanian triatomines must control the degree of habitat alteration and for the availability of hosts as potential biases when assessing pathogen-vector-host interactions.

*Rhodnius pallescens* and *T. dimidiata* are both primary vectors of *T. cruzi* in Panama, but the former seems more important from an epidemiologic standpoint due to its greater degree of association with *Attalea butyracea* in rural communities of Panama [35, 40, 85, 98]. Rural workers, “campesinos,” in these settings use leafs (Pencas) of *A. butyracea* to assemble the roof of their shacks [76], which might expedite invasion and also contribute to the spread of eggs, nymphs, and/or adults of *R. pallescens*. As a result, its genetic diversity is elevated by favoring gene flow among distantly located geographical populations [72]. *Triatoma dimidiata*, in contrast, is the primary vector of *T. cruzi* under more sylvatic conditions, in woody areas of Panama, where human settlements are established inside old-growth or secondary forest patches, and its populations reach large numbers regardless of the absence of *A. butyracea* [22, 45]. A trend of increasing forest degradation, suburbanization, and development of tourism in Panama indicates that a colonist species (i.e. disturbance tolerant) such as *R. pallescens* will continue to play a more prominent role in the transmission of *T. cruzi* than a forest specialist (i.e. disturbance intolerant) like *T. dimidiata* (Fig. 1). Other biologic attributes of *R. pallescens* supporting this view are high physiological plasticity, notable flight range by *T. cruzi*-infected individuals, and relatively short developmental time [40, 87, 102, 105].

### Taxonomic status

*Triatoma dimidiata* and *R. pallescens* distribute extensively across the neotropics, but the former has a greater geographical distribution. *Triatoma dimidiata* most likely originated in northern Central America (i.e. Mexico and Guatemala) and colonized southward through Mesoamerica and northern South America, whereas *R. pallescens* originated in South America (i.e. Colombia and Ecuador) and colonized northward across Mesoamerica [108–110]. Both species experienced episodes of vicariance and secondary admixture in the past, and face considerable environmental variability across their ranges at present. Therefore, they depict substantial phenotypic variance in color, size, behavior, and various levels of molecular divergence in mitochondrial and nuclear loci [105, 109, 111]. Several lines of evidence support the existence of at least three taxa within *T. dimidiata* (*s*.*l*.) (i.e. cryptic species complex), including *T. dimidiata capitata*, which is found in Panama and Colombia. Molecular divergence in *T. dimidiata* has been attributed to geographical range expansion following adaptation to local climatic conditions during its colonization of South America (e.g. Pleistocene climatic changes) or to more recent anthropogenic habitat degradation [108, 109, 112]. Likewise, *R. pallescens* depicts significant population structure across Panama, Colombia and Ecuador [110]. Two molecular lineages and a putative sympatric sibling species, *Rhodnius colombiensis*, were predicted to occur between northern South America and Panama. *Rhodnius pallescens* may be a complex of two isomorphic species with different chromosomal attributes. Values of molecular divergence between lineages I and II of *R. pallescens* were similar to those between *R. colombiensis* and these lineages, suggesting a very close phylogenetic relationship and perhaps similar ecologic niche among these three sister taxa (e.g. sylvatic habitat). Diversification of *R. pallescens* (lineages I and II) was attributed to the formation of the Isthmus of Panama, vicariance, and subsequent range re-colonization [110]. Studies about the population genetic structure and taxonomic status of *R. pallescens* and *T. dimidiata capitata* (i.e. the presence of additional cryptic evolutionary units) have not been conducted systematically across Panama, despite their potential to inform about the applicability of genetic vector control strategies.

### Prevention and control

Despite the ongoing expansion of CHD throughout ecologically altered areas of Panama, the Panamanian Ministry of Health (MINSA) does not consider this infection a priority in terms of control, limiting mitigation efforts to treat severe cases detected mostly by passive surveillance. Although the inattention to CHD by MINSA is likely due to the enzootic epidemiologic characteristic and chronic nature of CHD [2], some researchers attribute it to a significant degree of underreporting, and to the non-domiciliary behavior of *R. pallescens* in Panama [101, 103]. For instance, Panamanian populations of *R. pallescens* are highly susceptible to deltamethrin and lambda-cyhalothrin, but it is impractical to implement pyrethroid residual spraying to kill a vector that does not reside indoors [113]. CHD prevention and control programs in Panama must focus on putting into action an active surveillance program for accurate case detection. This program must also increase epidemiologic surveillance into unexplored areas of the countryside to detect new transmission foci [44, 45]. Recent work directed at CHD in Panama has highlighted the need to implement interdisciplinary approaches to prevent transmission, taking into account local changes in disease patterns due to anthropogenic and/or climatic changes, but actively involving community members in mitigation actions [2, 107]. The implementation of educational programs targeting vulnerable communities can help to minimize risk by teaching people how to improve house quality using appropriate construction materials, thus helping them to reduce human-vector contact [107, 114].

### Future research agenda

Once an active surveillance program is put into place and underreporting of *T. cruzi* is no longer an issue, forthcoming research about CHD in Panama must center on identifying epidemiologic risk factors in endemic areas, including ecologic (i.e. landscape uses affecting major vectors and reservoirs of CHD), demographic (i.e. gender and age range in human populations), environmental (i.e. temperature, precipitation, forest cover, and seasonality) and social variables (i.e. level of poverty and occupation) that may be related to a higher infection risk under certain conditions. Moreover, spatial and temporal clusters of CHD must be defined using hotspot analysis, geographical information system (GIS) mapping techniques, or landscape genetics as having been done recently for other vector-borne diseases in Panama [115]. Modeling the impact of climate change and/or forest alteration on the prevalence of *T. cruzi* can help to prevent future outbreaks. A recent study demonstrated the utility of the macro-ecological approach to better understand the spatial-temporal transmission dynamic of leishmaniasis in Panama [116], but similar work on CHD has not yet been conducted. In addition, there is a need for developing new and innovative strategies for vector control and more effective and less toxic drugs to treat infected people [72, 74]. More specifically, scientific studies about population genetics and niche dynamics of major epidemiologic components of CHD (i.e. parasite-vector-host) are lacking in Panama. The taxonomic status of *T. dimidiata capitata* and *R. pallescens* must be further evaluated, as well as possible differences in vector bionomic or insecticide resistance profiles among different subpopulations (e.g. lineages or sister taxa) of these vectors [117]. In addition, no systematic study of the distribution of vector and non-vector triatomines or about inter-specific competition among triatomine species has ever been conducted in the country. Tourism is growing in Panama, and certain real estate developments use *A. butyracea* and other species of palm trees (i.e. *Acrocomia* sp.) for esthetic purposes, which could potentially open new niches for some triatomine species [98]. It is also necessary to investigate the role of recurrent infections with *T. rangeli* on CHD transmission in Panama because this sympatric parasite greatly decreases the fitness of *R. pallescens*. These and other topics must be tackled to understand the evolutionary potential of CHD under an increasing scenario of climate change and urbanization.

## Conclusions

Extensive ecological and epidemiological investigation about CHD has been undertaken in Panama since the beginning of last century, and several generations of Panamanian scientists have been involved in these efforts. Furthermore, lately there has been a growing interest in investigating CHD in Panama, and a new theoretical approach linking anthropogenic degradation of forest ecosystems with CHD emergence has been implemented. However, very few attempts have been made in Panama to integrate all this information into prevention and mitigation actions for CHD control [118–120]. The first logical step in planning effective strategies to prevent and manage CHD expansion across the country is to summarize existing information on scientific research. Here we make progress toward this end, reviewing knowledge about the ecology and epidemiology of CHD since the 1930s. Seemingly easy and yet challenging at once, this task is often neglected in countries with a rich history of research in tropical medicine, where past scientific information is no longer read by newer generations of scientists and might be scattered or lost [118, 120]. This might be the case in Panama, where no review article has ever been written in English about CHD, despite the rich history of scientific investigations on this neglected vector-borne infection. As a philosophical conclusion, we posit that scientific research about CHD must continue in Panama, and will prove to be the best weapon to lessen transmission risk in endemic areas. However, the government should make it a public health priority and establish an effective active surveillance program as the first step to mitigating this problem [107, 118, 120]. Finally, future research plans about CHD in Panama must continue using community ecology and hypothesis testing as the primary instruments to understand the overlooked complexity of this disease system better. For now, this is a more realistic approach than the pattern of discovery/data reporting type of research, and it will result in the generation of valid scientific information that could be used to design integrated and effective mitigation strategies. We hope that our review article will contribute to the very first step of this crucial goal, as it provides a summary of information on ecologic and epidemiologic research, which along with knowledge about the impact of anthropogenic habitat alterations and climate change into CHD transmission will help to diminish the burden of this neglected tropical infection in Panama.

## Additional file

Additional file 1: Table S1. References concerning the ecology and epidemiology of American trypanosomiasis or Chagas Disease, in Panama. (XLSX 497 kb)

## Abbreviations

CHD: Chagas disease
CTFS: Center for Tropical Forest Science
DNA: Deoxyribonucleic acid
DTU: Discrete typing units
GIS: Geographical information system
INDICASAT-AIP: Instituto de Investigaciones Científicas y Servicios de Alta Tecnología
MINSA: Panamanian Ministry of Health
PCR: Polymerase chain reaction
SNI: National System of Investigation
STRI: Smithsonian Tropical Research Institute
